# Tracking of menstrual cycles and prediction of the fertile window via measurements of basal body temperature and heart rate as well as machine-learning algorithms

**DOI:** 10.1186/s12958-022-00993-4

**Published:** 2022-08-13

**Authors:** Jia-Le Yu, Yun-Fei Su, Chen Zhang, Li Jin, Xian-Hua Lin, Lu-Ting Chen, He-Feng Huang, Yan-Ting Wu

**Affiliations:** 1 The International Peace Maternity and Child Health Hospital, School of Medicine, Shanghai Jiao Tong University, Shanghai, China; 2 Shanghai Key Laboratory of Embryo Original Diseases, Shanghai, China; 3 Shanghai Municipal Key Clinical Speciality, Shanghai, China; 4 Obstetrics and Gynecology Hospital, Institute of Reproduction and Development, Fudan University, No. 419, Fangxie Rd, Shanghai, 200011 China; 5 Research Units of Embryo Original Diseases, Chinese Academy of Medical Sciences (No. 2019RU056), Shanghai, China; 6 Key Laboratory of Reproductive Genetics (Ministry of Education), Department of Reproductive Endocrinology, Women’s Hospital, Zhejiang University School of Medicine, Hangzhou, China

**Keywords:** Wearable device, Machine learning, Fertile window, Menstrual cycle, Heart rate, Basal body temperature

## Abstract

**Background:**

Fertility awareness and menses prediction are important for improving fecundability and health management. Previous studies have used physiological parameters, such as basal body temperature (BBT) and heart rate (HR), to predict the fertile window and menses. However, their accuracy is far from satisfactory. Additionally, few researchers have examined irregular menstruators. Thus, we aimed to develop fertile window and menstruation prediction algorithms for both regular and irregular menstruators.

**Methods:**

This was a prospective observational cohort study conducted at the International Peace Maternity and Child Health Hospital in Shanghai, China. Participants were recruited from August 2020 to November 2020 and followed up for at least four menstrual cycles. Participants used an ear thermometer to assess BBT and wore the Huawei Band 5 to record HR. Ovarian ultrasound and serum hormone levels were used to determine the ovulation day. Menstruation was self-reported by women. We used linear mixed models to assess changes in physiological parameters and developed probability function estimation models to predict the fertile window and menses with machine learning.

**Results:**

We included data from 305 and 77 qualified cycles with confirmed ovulations from 89 regular menstruators and 25 irregular menstruators, respectively. For regular menstruators, BBT and HR were significantly higher during fertile phase than follicular phase and peaked in the luteal phase (all *P* < 0.001). The physiological parameters of irregular menstruators followed a similar trend. Based on BBT and HR, we developed algorithms that predicted the fertile window with an accuracy of 87.46%, sensitivity of 69.30%, specificity of 92.00%, and AUC of 0.8993 and menses with an accuracy of 89.60%, sensitivity of 70.70%, and specificity of 94.30%, and AUC of 0.7849 among regular menstruators. For irregular menstruators, the accuracy, sensitivity, specificity and AUC were 72.51%, 21.00%, 82.90%, and 0.5808 respectively, for fertile window prediction and 75.90%, 36.30%, 84.40%, and 0.6759 for menses prediction.

**Conclusions:**

By combining BBT and HR recorded by the Huawei Band 5, our algorithms achieved relatively ideal performance for predicting the fertile window and menses among regular menstruators. For irregular menstruators, the algorithms showed potential feasibility but still need further investigation.

**Trial registration:**

ChiCTR2000036556. Registered 24 August 2020.

**Supplementary Information:**

The online version contains supplementary material available at 10.1186/s12958-022-00993-4.

## Introduction

Accurate menstrual cycle tracking and identification of the fertile window are in high demand worldwide for determining the optimal time of intercourse among couples who desire to conceive. Wilcox et al. were among the first to identify the fertile window, the most fecund period of the menstrual cycle, which is comprised of the five days preceding ovulation and the day of ovulation [1]. Many biomarkers, including changes in basal body temperature (BBT), vaginal discharge, the position of cervix, and luteinizing hormone (LH) levels (which can be assessed in home urine tests), are used to detect ovulation and the fertile window to aid conception; among these, monitoring of BBT is one of the most convenient and least invasive [2]. The nadir (the lowest point) of BBT occurs approximately one day before ovulation [2]. In response to elevated levels of progesterone, BBT rises significantly approximately 2 days after the LH peak [3]. According to a cohort study, 21% of women have used BBT measurements to track their fecundity [4]. However, studies have shown that BBT measurements are not able to precisely predict the fertile window [5, 6]. In addition, traditional BBT records can only retrospectively confirm the fertile window by providing evidence of ovulation 2 or 3 days after its occurrence [3]. Even though there may be a BBT nadir before ovulation, this change does not feasibly predict the fertile window since not all nadirs coincide with ovulation [6].

Over the past few years, the use of websites and cellular phone applications (apps) to track menstrual cycles and predict the ovulation day or fertile window has become increasingly popular [7]. These apps are based on records of BBT, cervical fluid, cervix position and urine LH levels [8]. However, despite their convenience, the accuracy of their fertile window predictions is generally far from satisfactory. Current applications based on BBT have low prediction accuracy for ovulation since the algorithm is dependent on BBT data or previous user cycles [9]. Thus, more advanced app-based technology is urgently needed to aid couples who desire to conceive.

Advances in portable sensors and wearable technology provide continuous dynamic health information over the course of a day. In addition, machine learning based on health data has been increasingly applied to clinical medicine, including the field of fertility awareness. Pairing machine learning with health data from wearable devices has the potential to improve the practice of clinical medicine. Some studies have already used machine learning to predict menstruation, ovulation and the fertile window based on body temperature data recorded by wearables [10]. Since previous studies have shown that heart rate (HR) varies during different phases of the menstrual cycle, with a higher rate during ovulation [11], some studies have employed wearables that collect data on body temperature, HR, heart rate variability (HRV), respiratory rate, etc., to develop algorithms for the prediction of ovulation and the fertile window [12]. However, most of these studies failed to specifically focus on women with irregular menstrual cycles [13, 14]. Little is known about whether physiological data collected by wearables can be used to track menstrual cycles and predict the fertile window among women with irregular menstrual cycles. Furthermore, few studies have focused on the menstruation period of women with irregular cycles.

In this study, we aimed to evaluate alterations in BBT (measured with an ear thermometer) and HR (recorded by a wearable device, the Huawei Band 5) during different phases in the menstrual cycle. In addition, we further developed machine-learning algorithms that integrated BBT and HR data to predict the fertile window and menstruation days among women with regular menstrual cycles. Finally, we explored the feasibility of fertile window and menstruation day prediction among women with irregular menstrual cycles.

## Methods

### Study design and participants

This was a prospective observational cohort study conducted at the International Peace Maternity and Child Health Hospital (IPMCHH) in Shanghai, China. Participants were recruited from August 2020 to November 2020 and followed up between August 2020 and May 2021. Eligible participants were nonpregnant women aged 18–45 with natural menstrual cycles. For this study, we excluded women who had major diseases, had a history of pregnancy within the past 6 months, were breastfeeding, were taking medications that could interfere with the menstrual cycle (e.g., hormonal contraception or hormone replacement), traveled across time zones during the follow-up period or had a sleeping disorder. We divided women by menstrual cycle length, which was defined as the time in days from the first day of one menses to the first day of the next [15]. Since self-reported menstrual cycle length is proven reliable [15], women were queried about the usual length of the menstrual cycle without hormonal therapies in the past year [16–18]. Women with usual menstrual cycle lengths of 25–35 days were included in the regular group. Women with usual menstrual cycle lengths outside of this range were included in the irregular group [19, 20].

We recruited women via advertisements on WeChat APP of doctors at the IPMCHH. At enrollment, participants completed baseline questionnaires with items on age, weight, height, marital status, educational attainment, occupation, age at menarche, smoking status, alcohol consumption, and history of pregnancy and childbirth. The participants also received a wearable sensor (Huawei Band 5; Huawei Device Co, Ltd, Shenzhen, China), a smartphone (Huawei Mate 30; Huawei Device Co, Ltd, Shenzhen, China) and an ear thermometer (Braun IRT6520) to record essential physiological data. Participants were followed up at least four menstrual cycles and encouraged to retain in the cohort until they had completed 4 qualified menstrual cycles, which was defined as synced data for 80% of the cycle durations. Women were gifted the study materials (i.e., the sensor, smartphone, and thermometer) at the end of their follow-up.

### Study protocol

#### Data collection

During the follow-up period, women were asked to wear the Huawei Band 5 every night while sleeping and to sync this data with their smartphones every morning. For data collection, the duration of continuous sleep had to exceed 4 h every night. The Huawei Band 5 measures HR and HRV continuously during sleep. It can also measure data related to sleep quantity and sleep quality. Since our research did not focus on sleep, these data were not included in the analysis. BBT was assessed daily with an ear thermometer in the morning upon waking, after participants had lain horizontally for 5 min. Participants were instructed to report menstruation on the smartphone by answering “yes” or “no” to the two questions (i.e., “Did your period start/end today?”) every day.

#### Determination of ovulation

Ovulation was determined by an ultrasound and detection of serum hormone levels [21]. In the first cycle, participants underwent transvaginal or abdominal ultrasound from cycle day 8–12 to the day that a follicle reached 17 mm [22]. Serum hormone levels, including LH, estradiol (E2), follicle-stimulating hormone (FSH), progesterone, testosterone, prolactin, and anti-Müllerian hormone (AMH), were measured on the day of large (i.e., at least 17 mm) follicle detection. According to hormone levels, another ultrasound was performed several days later to confirm ovulation (follicular enlargement followed by evidence of rupture). In cases where follicular enlargement was missed, the progesterone level was assayed. Based on the day of maximum follicular enlargement and levels of LH, E2, and progesterone as well as evidence of rupture, the day of ovulation was estimated.

During the next three menstrual cycles, the first day of follow-up was determined by a senior gynecologist according to the ovulation day in the first cycle. Basically, it would be one or two days before the previous ovulation day. All monitoring procedures were the same as those in the first cycle except that the blood samples were analyzed only for levels of LH, E2, FSH and progesterone. In the present study, data of cycles without determined ovulation were excluded.

According to the days of menstruation and ovulation, the menstrual cycle was divided into 4 phases. The duration of menstrual flow was defined as the menstrual phase reported by each woman [23]. The follicular phase (outside menses and the fertile window) was the time between the first day post-menses and 6 days before ovulation [24]. The fertile phase lasted from 5 days before ovulation to the ovulation day [1, 19, 25]. The luteal phase started post-ovulation and lasted until the day before menses [24].

### Statistical analysis

Participant characteristics are presented with descriptive statistics. Continuous variables that were not normally distributed are displayed as medians and interquartile ranges. These variables were compared with the Wilcoxon test. Categorical variables are expressed as frequencies with proportions, with group differences assessed with the chi-square test and Fisher’s exact test.

Before analyzing the synced data from the wearable sensors, we excluded data collected during the first 30 min after sleep and the last 30 min before waking. HR was extracted from the photoplethysmographic signal. HRV indices, including the standard deviation of normal-to-normal intervals (SDNN) values and the low frequency (LF)/high frequency (HF) ratio, were obtained by analyzing R-R interval recordings. Missing values were replaced by the mean of values from two days before and after the day with missing data.

Data on qualified menstrual cycles with identified ovulation days were included in the analysis. Normality tests [26] indicated that physiological parameters BBT and HR were normally distributed, but SDNN and LF/HF ratio were skewed. Thus, SDNN and LF/HF ratio were natural log transformed (ln) to improve normality [27]. We did further analysis on the transformed data. Each participant contributed data from several menstrual cycles, which created a nested structure in our data. To estimate the effect of cycle phase on physiological parameters, we used a linear mixed model that estimated fixed and random effects [28] (R packages: lme4 and lmerTest). We specified *cycle phase* as a fixed effect and *participants/menstrual cycle* as a random intercept in the model. For multiple comparisons, the Bonferroni method was used to adjust the *P* value (R package: multcomp). We then set the *group* as a fixed effect and ran a series of models as mentioned above to investigate the group differences in physiological parameters. We tested all linear mixed models by examining their residuals.

All data analyses and visualizations were performed with R software (version 4.0.2, R Foundation for Statistical Computing, Vienna, Austria). A two-sided *P* value of less than 0.05 was considered statistically significant.

### Construction of the algorithms

According to best practices, BBT and HR waveforms were smoothed at the single cycle level. From the changes and analysis of physiological signs, biphasic rule and features were extracted from both waveforms and the fertile day was most likely located on the switch point of the waveforms. SDNN and LF/HF ratio were preprocessed in a similar way. More details were provided in supplementary methods. Based on the analysis and data preparation, we developed a probability function estimation model to estimate the probability of whether a day fell in the fertile window. Specifically, to estimate whether Day 1 was in the fertile window, data from the last consecutive period before Day 1 were used for model input. The construction of the menstruation prediction model is similar to that of the fertile window prediction model.

The dataset of enrolled patients was partitioned randomly into training and testing groups. In detail, data from 225 qualified cycles from 68 participants in the regular group were used as the training dataset; 80 qualified cycles from 21 participants in the regular group and 77 cycles from 25 participants in the irregular group were included in the two testing datasets respectively. Based on a training dataset, a fivefold cross validation method was used to determine the model. Firstly, the training data was divided into 5 parts. In every iteration, 4 parts were aggregated as training subsets and the last part as a testing subset. Then the hyperparameters were optimized and adjusted, and the model was preliminarily evaluated. Finally, the model performance was evaluated with the testing dataset. We calculated the accuracy, sensitivity, specificity, and area under the receiver operating characteristic (ROC) curve (AUC) to measure the algorithm performance. The algorithms were developed and visualized using Python software (version 3.10.4, The Python Software Foundation, USA).

## Results

**Fig. 1:**
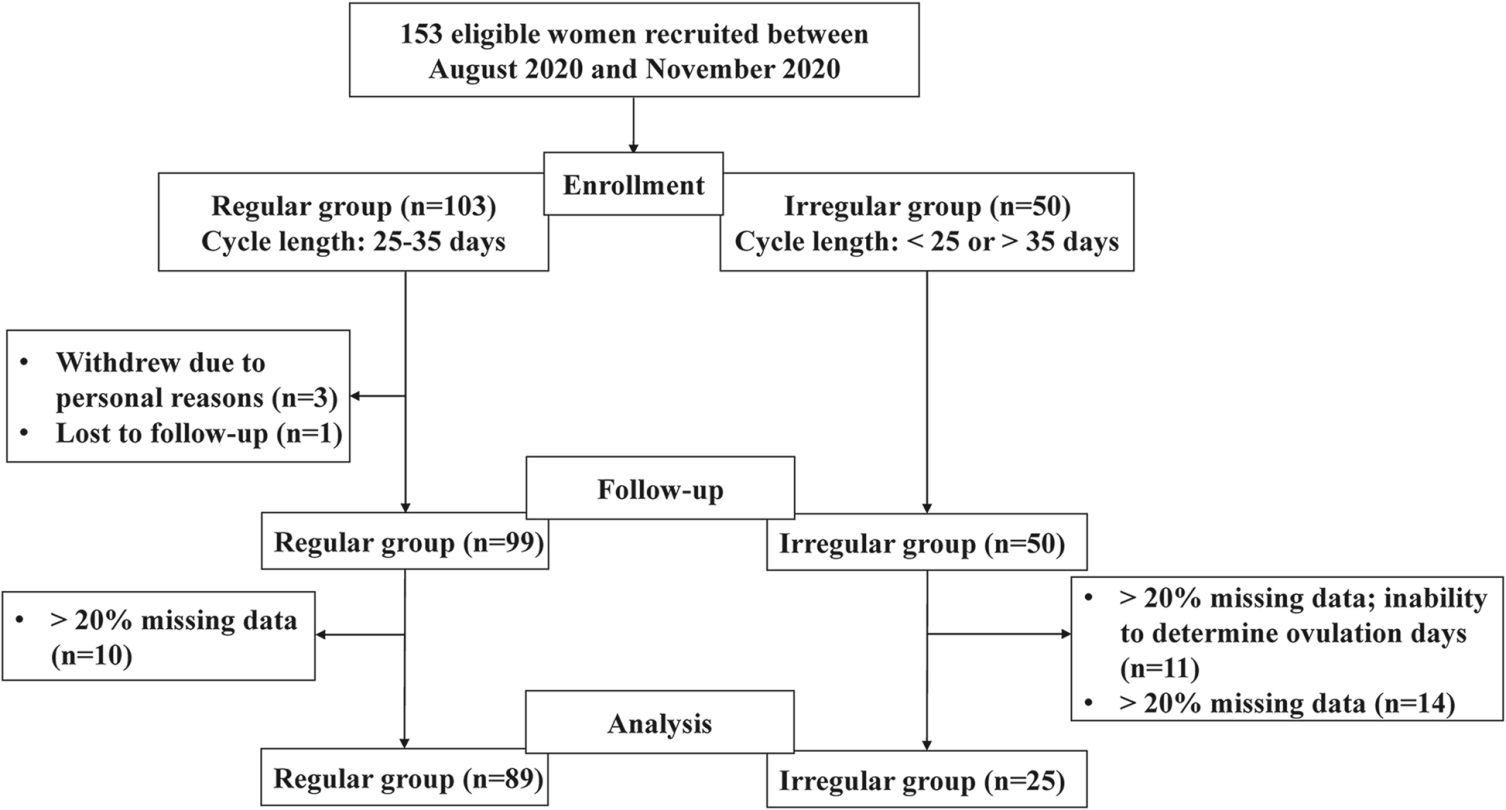
Flow chart of the study population

A total of 153 eligible women with regular menstrual cycles (*n* = 103) or irregular menstrual cycles (*n* = 50) were initially recruited for this study (Fig. 1). However, 3 women in the regular group withdrew early because they were no longer willing to participate, and one participant was lost to follow-up after the first cycle. Therefore, 149 women eventually completed all procedures. Of these participants, 10 women in the regular group and 14 women in the irregular group were excluded due to > 20% missing data. In addition, 11 women in the irregular group were excluded from the final analysis due to > 20% missing data and inability to determine ovulation day. Thus, by the end of data collection, 89 women in the regular group and 25 women in the irregular group completed qualified menstrual cycles, synced data on which was 80% complete and which had determined ovulation days. Data from these 114 women were included in the final analysis.

The characteristics of all participants are summarized in Table S1. Table 1 presents the characteristics of the women included in the final analysis. Women in the irregular group were younger than those in the regular group (*P* = 0.005). However, body mass index (BMI), marital status, educational attainment, occupation, duration of menstruation, age at menarche, smoking status, alcohol consumption, and history of pregnancy and childbirth were comparable between the two groups. The AMH levels significantly differed between the two groups (*P* = 0.035); the irregular group was more likely to have a low AMH (< 0.7 ng/ml) (12.0% vs. 4.5%) or high AMH (> 7.5 ng/ml) (28.0% vs. 12.4%) than the regular group. During the entire follow-up period, women in the regular group had cycles with a median length of 29 (interquartile range [IQR] = 28–32) days, while women in the irregular group had a median cycle length of 33 (IQR = 27–40) days.

**Table 1:** Characteristics of participants included in the analysis

	**All**	**Regular**	**Irregular**	***P***
	**(*N* = 114)**	**(*N* = 89)**	**(*N* = 25)**	
	**No. (%)**	**No. (%)**	**No. (%)**	
**Age, median (IQR** ^a^**), years**	31.00 (26.00, 34.00)	32.00 (27.00, 35.00)	28.00 (25.00, 31.00)	0.005
**Age groups, years**				0.017
18–24	14 (12.3)	8 (9.0)	6 (24.0)	
25–30	37 (32.5)	25 (28.1)	12 (48.0)	
31–35	43 (37.7)	38 (42.7)	5 (20.0)	
36–45	20 (17.5)	18 (20.2)	2 (8.0)	
**BMI** ^b^**, median (IQR** ^a^**), kg/m**^**2**^	20.73 (19.30, 22.67)	20.82 (19.33, 22.59)	20.70 (19.29, 23.51)	0.88
**BMI groups**				0.294
< 18.5	12 (10.5)	8 (9.0)	4 (16.0)	
18.5–23.9	86 (75.4)	70 (78.7)	16 (64.0)	
≥ 24	16 (14.0)	11 (12.4)	5 (20.0)	
**Marital status**				0.42
Married	54 (47.4)	45 (50.6)	9 (36.0)	
Single	59 (51.8)	43 (48.3)	16 (64.0)	
Divorced	1 (0.9)	1 (1.1)	0 (0.0)	
**Educational attainment**				0.599
High school	4 (3.5)	4 (4.5)	0 (0.0)	
College	52 (45.6)	39 (43.8)	13 (52.0)	
Master’s or above	58 (50.9)	46 (51.7)	12 (48.0)	
**Occupation**				0.069
Unemployed	29 (25.4)	20 (22.5)	9 (36.0)	
Full-time worker	84 (73.7)	69 (77.5)	15 (60.0)	
Part-time worker	1 (0.9)	0 (0.0)	1 (4.0)	
**Duration of menstruation, median (IQR** ^a^**), days**	6.00 (5.00, 7.00)	6.00 (5.00, 7.00)	6.00 (5.00, 7.00)	0.319
**Menarche, median (IQR** ^a^**), years**	13.00 (12.00, 14.00)	13.00 (12.00, 14.00)	13.00 (12.00, 14.00)	0.699
**Smokers**	3 (2.6)	3 (3.4)	0 (0.0)	1
**Consume alcohol**	27 (23.7)	24 (27.0)	3 (12.0)	0.197
**Previous pregnancy**	49 (43.0)	43 (48.3)	6 (24.0)	0.052
**Previous childbirth**	49 (43.0)	43 (48.3)	6 (24.0)	0.052
**AMH** ^c^**, median (IQR** ^a^**), ng/ml**	3.90 (2.13, 5.61)	3.53 (2.09, 5.25)	4.84 (3.24, 7.73)	0.062
**AMH** ^c^ **groups**				0.035
< 0.7	7 (6.1)	4 (4.5)	3 (12.0)	
0.7–7.5	89 (78.1)	74 (83.1)	15 (60.0)	
> 7.5	18 (15.8)	11 (12.4)	7 (28.0)	

^a^IQR, interquartile range

^b^BMI, body mass index

^c^AMH, Anti-Müllerian hormone

**Fig. 2:**
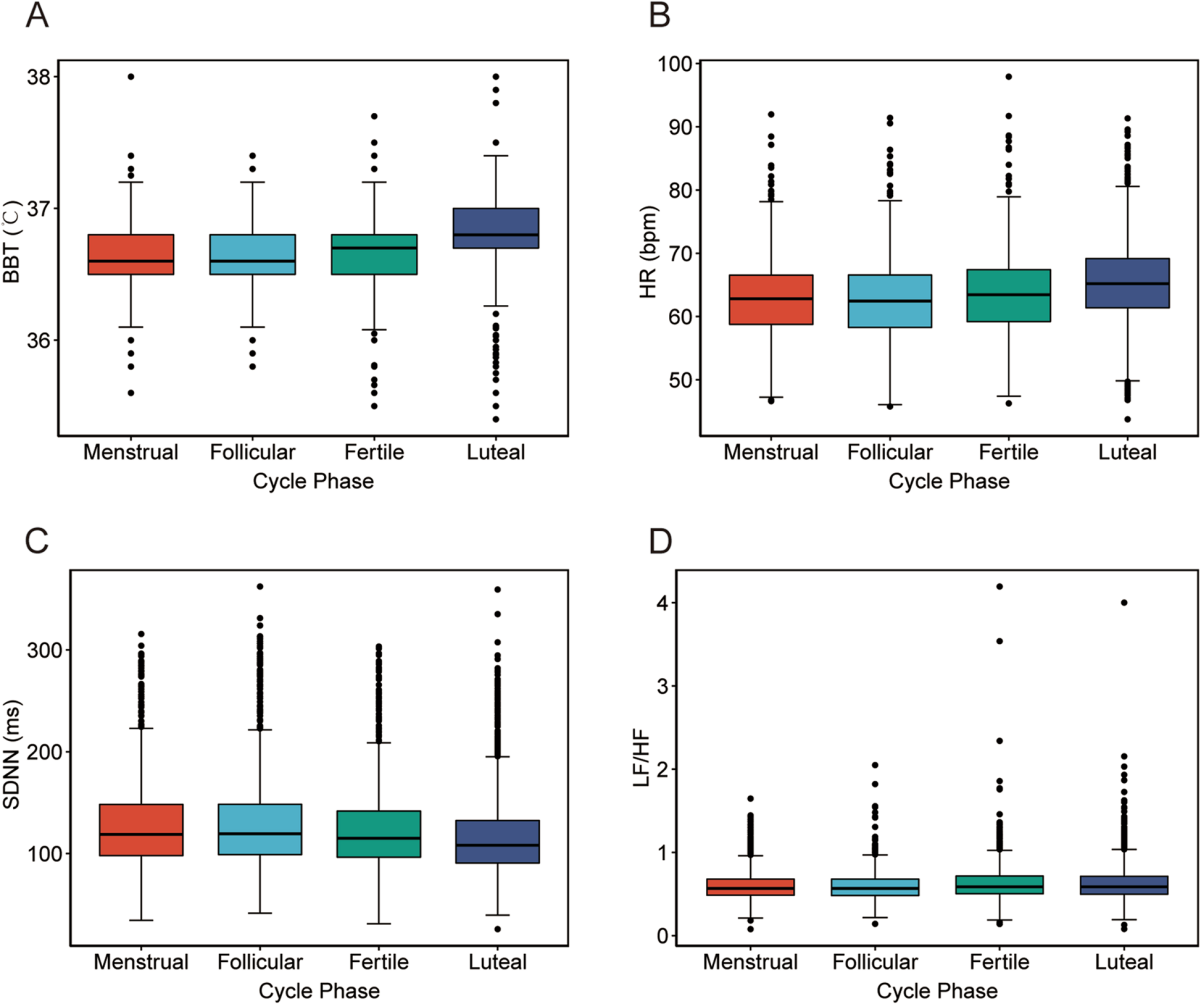
Physiological parameters of the regular group in the different phases of the menstrual cycle. Changes in BBT (**A**), HR (**B**), SDNN (**C**) and the LF/HF ratio (**D**) during the menstrual cycle of regular menstruators are depicted. The horizontal line represents the medians, boxes represent the values between 25–75%, and lines represent the values between 5–95%. BBT: basal body temperature; HR: heart rate; LF/HF: low frequency /high frequency ratio; SDNN: standard deviation of normal-to-normal intervals

The physiological parameters changed regularly throughout the menstrual cycle (Fig. 2 and Figure S1). For regular menstruators (Table 2), BBT was significantly lower in the follicular phase than in the menstrual phase (*P* < 0.001). In the fertile phase, BBT was 0.04 ℃ higher than in the follicular phase (*P* < 0.001). BBT in the luteal phase increased 0.18 ℃ compared with that in the fertile phase (*P* < 0.001). Unlike BBT, HR, ln(SDNN) and ln(LF/HF ratio) in the follicular phase were similar to those in the menstrual phase. Consistent with BBT, HR in the fertile phase was significantly higher (0.61 bpm; *P* < 0.001) than that in the previous stage. Furthermore, HR peaked in the luteal phase, increasing by 1.84 bmp (*P* < 0.001) compared with the fertile phase. In contrast to HR, ln(SDNN) in the fertile phase was lower (-0.04; *P* < 0.001) than that in the follicular phase. Ln(SDNN) reached its lowest value in the luteal phase. Compared to that in the follicular phase, ln(LF/HF ratio) in the fertile phase increased 0.03 (*P* < 0.001); this increase was maintained in the luteal phase. Throughout the whole cycle, the fertile phase always was the "inflection point" for the physiological parameters.

**Table 2:** The relationship between menstrual cycle and physiological parameters in the regular group

Physiological parameter	BBT^a, b^	HR^a, c^	Ln(SDNN^a, d^)	Ln(LF/HF ratio^a, e^)
Intercept	36.63 (0.02)	62.50 (0.54)	4.80 (0.02)	-0.55 (0.02)
**Cycle phase**				
Menstrual	Reference	Reference	Reference	Reference
Follicular	-0.03 (0.01)^f^	0.23 (0.13)	-0.00 (0.01)	0.02 (0.01)
Fertile	0.01 (0.01)	0.84 (0.12)^f^	-0.04 (0.01)^f^	0.05 (0.01)^f^
Luteal	0.19 (0.01)^f^	2.69 (0.11)^f^	-0.10 (0.01)^f^	0.05 (0.01)^f^
Follicular	Reference	Reference	Reference	Reference
Fertile	0.04 (0.01)^f^	0.61 (0.13)^f^	-0.04 (0.01)^f^	0.03 (0.01)^f^
Fertile	Reference	Reference	Reference	Reference
Luteal	0.18 (0.01)^f^	1.84 (0.11)^f^	-0.06 (0.01)^f^	0.00 (0.01)

^a^Unstandardized beta-coefficient values (standard error) reported with adjusted *p* values using a Bonferroni correction

^b^BBT, basal body temperature

^c^HR, heart rate

^d^SDNN, standard deviation of normal-to-normal intervals

^e^LF/HF ratio, low frequency /high frequency ratio

^f^*P* < 0.001

The trend of changes in physiological parameters in the irregular group throughout the menstrual cycle was similar to that in the regular group (Table S2). For menstrual cycles with an ovulation day, the physiological parameters of all phases were similar between the regular and irregular groups (Table 3).

**Table 3:** Comparison of physiological parameters between the regular and irregular groups

Physiological parameter	Cycle phase	Menstrual^a^	Follicular^a^	Fertile^a^	Luteal^a^
**BBT**^b^	Intercept	36.64 (0.02)	36.61 (0.02)	36.64 (0.02)	36.82 (0.02)
	**Group**				
	Regular	Reference	Reference	Reference	Reference
	Irregular	-0.04 (0.05)	-0.04 (0.04)	-0.07 (0.05)	-0.05 (0.05)
**HR**^c^	Intercept	62.56 (0.58)	62.64 (0.58)	63.31 (0.58)	65.12 (0.59)
	**Group**				
	Regular	Reference	Reference	Reference	Reference
	Irregular	-0.01 (1.24)	-0.20 (1.23)	0.19 (1.24)	0.75 (1.26)
**Ln(SDNN**^d^)	Intercept	4.80 (0.03)	4.80 (0.03)	4.77 (0.02)	4.71 (0.02)
	**Group**				
	Regular	Reference	Reference	Reference	Reference
	Irregular	-0.06 (0.05)	-0.06 (0.06)	-0.06 (0.05)	-0.06 (0.05)
**Ln(LF/HF ratio**^e^)	Intercept	-0.55 (0.02)	-0.54 (0.02)	-0.50 (0.02)	-0.51 (0.02)
	**Group**				
	Regular	Reference	Reference	Reference	Reference
	Irregular	-0.02 (0.05)	-0.02 (0.05)	-0.02 (0.05)	-0.00 (0.05)

^a^Unstandardized beta-coefficient values (standard error) are reported

^b^BBT, basal body temperature

^c^HR, heart rate

^d^SDNN, standard deviation of normal-to-normal intervals

^e^LF/HF ratio, low frequency /high frequency ratio

**Fig. 3:**
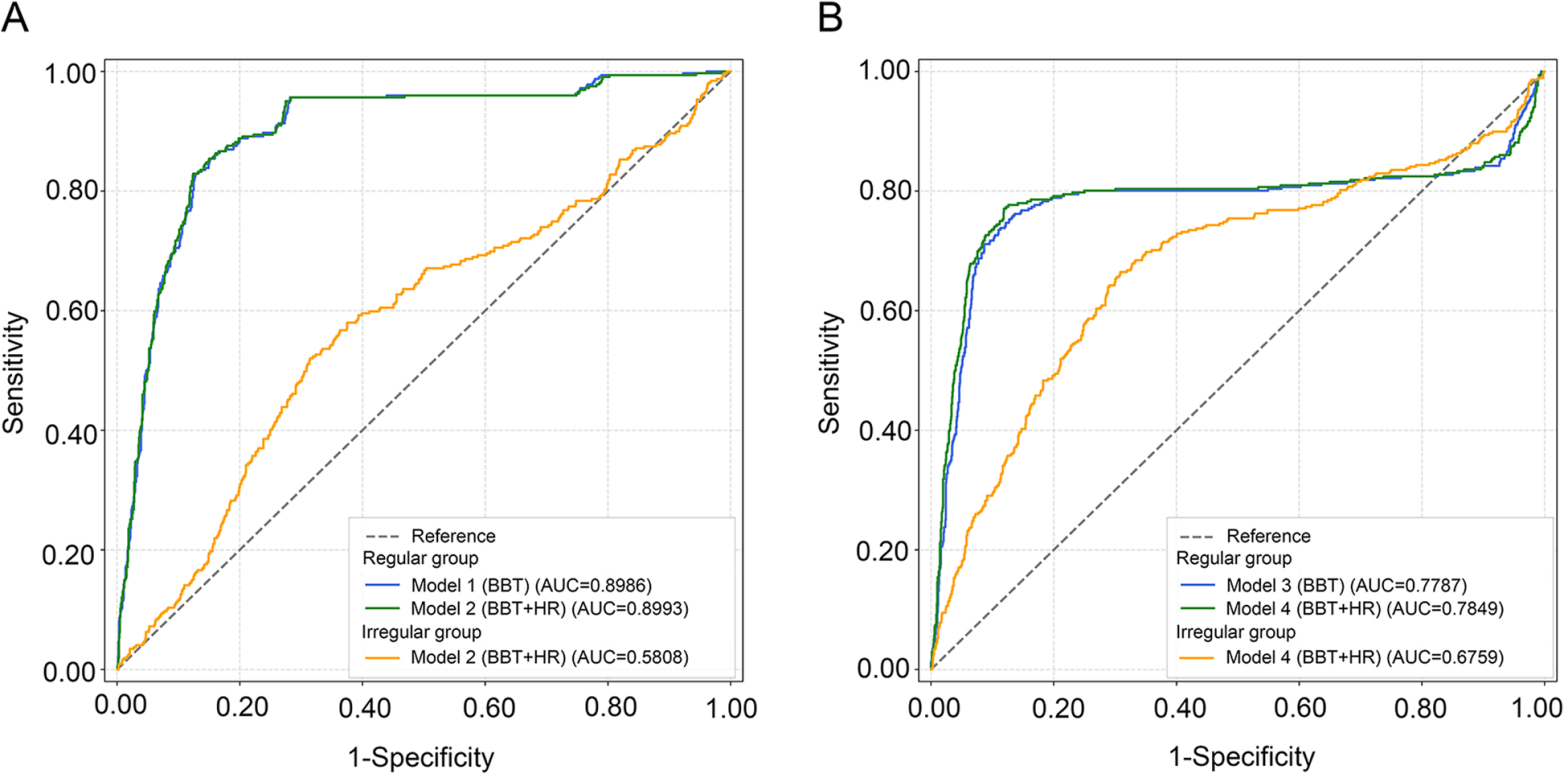
Prediction performance of different models. ROC curves of fertile window (**A**) and menstruation (**B**) prediction models based on BBT and HR for regular and irregular groups. AUC: area under the receiver-operating characteristic curve; BBT: basal body temperature; HR: heart rate; ROC: receiver operating characteristic

We developed a series of predictive models (Table 4) for detecting the fertile window and menstruation using data from 114 women, including 305 qualified cycles with ovulation from 89 participants in the regular group and 77 qualified cycles with ovulation from 25 participants in the irregular group. The algorithm based on the BBT of the regular group achieved an accuracy of 86.65%, sensitivity of 68.30% and specificity of 91.30% in predicting the fertile window of women with regular menstrual cycles. Furthermore, the prediction performance improved to an accuracy of 87.46%, sensitivity of 69.30% and specificity of 92.00% when integrating HR collected by the wearable Huawei Band 5. The algorithm developed from data from regular menstruators was also applied to predict the fertile window of irregular menstruators and achieved an accuracy of 72.51%, sensitivity of 21.00%, and specificity of 82.90%. The algorithm trained with the BBT data from regular menstruators predicted menses with an accuracy of 87.80%, sensitivity of 66.10% and specificity of 93.10% in women with regular menstrual cycles. When we integrated HR data into the model, the prediction performance improved to an accuracy of 89.60%, sensitivity of 70.70% and specificity of 94.30%. The algorithm was also applied to predict menses of irregular menstruators and achieved an accuracy of 75.90%, sensitivity of 36.30%, and specificity of 84.40% for menstrual phase prediction. The ROC curves and the AUCs of different models are provided in Fig. 3. The fertile window prediction models developed among regular menstruators using BBT and BBT combined with HR had AUCs of 0.8986 and 0.8993, respectively. The latter one was applied to predict the fertile window among irregular menstruators and achieved an AUC of 0.5808. The menstruation prediction models developed among regular menstruators based on BBT and BBT combined with HR had AUCs of 0.7787 and 0.7849, respectively. The latter one was applied to predict menses among irregular menstruators and achieved an AUC of 0.6759.

**Table 4:** Accuracy, sensitivity and specificity of different models in fertile window and menstruation prediction

Cycle phase	Prediction model (physiological parameter)	Accuracy (%)	Sensitivity (%)	Specificity (%)
**Fertile**	**Regular group**			
	Model 1 (BBT^a^)	86.65	68.30	91.30
	Model 2 (BBT ^a^ and HR^b^)	87.46	69.30	92.00
	**Irregular group**			
	Model 2 (BBT ^a^ and HR^b^)	72.51	21.00	82.90
**Menstrual**	**Regular group**			
	Model 3 (BBT^a^)	87.80	66.10	93.10
	Model 4 (BBT^a^ and HR^b^)	89.60	70.70	94.30
	**Irregular group**			
	Model 4 (BBT^a^ and HR^b^)	75.90	36.30	84.40

^a^BBT, basal body temperature

^b^HR, heart rate

To further compare the performance of the current model in predicting fertile window with other algorithms, we tried to integrate SDNN and LF/HF ratio data into the model and use the data from the irregular group for training (Table S3). Overall, the models developed using four features had similar performance with the model based on BBT and HR. Among models based on BBT and HR, the model trained using BBT and HR data from the regular group still achieved the best accuracy, sensitivity and specificity among regular menstruators. It achieved the same sensitivity (35.8%), a slightly lower accuracy (78.78% vs 77.31%) and specificity (87.60% vs 85.80%) among irregular menstruators compared to the model based on data of the irregular group. In conclusion, the model which included only BBT and HR based on data from participants with regular menstrual cycles provided relatively robust and stable performance for fertile window prediction among both regular and irregular menstruators.

In order to improve the performance of fertile window prediction among irregular menstruators, we divided them into two subgroups based on the cycle length. The current model had better predictive accuracy and sensitivity in women with cycle length > 35 days and better predictive specificity in women with cycle length < 25 days (Table S4). Additionally, the current model was more suitable for women over 35 years old in predicting the fertile window among both regular menstruators and irregular menstruators (Table S5).

## Discussion

### Principal findings

To the best of our knowledge, this is the first study to apply wearable technology and machine learning methods to predict the fertile window and menstruation days for women with regular and irregular cycles. Here, we demonstrated that 1) the in-ear temperature and HR collected by the Huawei Band 5 followed a similar pattern in women with regular and irregular menstrual cycles; 2) the algorithm that used BBT data and HR data from the Huawei Band 5 outperformed the algorithm that used BBT data alone in predicting the fertile window and menstruation of women with regular menstrual cycles in terms of accuracy, sensitivity, specificity and AUC; 3) the fertile window and menstruation of women with irregular menstrual cycles can potentially be predicted using the algorithms but with lower accuracy, sensitivity, specificity and AUC.

According to the results, the tympanic membrane temperature during the luteal phase differed significantly from that of the menstrual phase, at approximately 0.2 ℃ higher; this difference was expected according to previous literature [3, 25]. Our finding of the pattern of changes in HR were consistent with some other studies that measured HR using electrocardiography. Matthew et al. examined HRV across the menstrual cycle in 13 women with normal menstruation, confirming an increase in HR and a decrease in ln(SDNN) after ovulation [11]. Similar to our findings, Bai et al. also found that the LF/HF ratio and HR increased from the follicular phase to the luteal phase [29]. These results demonstrate that data collected by the Huawei Band 5 were capable of reflecting phase-based changes in HR as accurately as an electrocardiogram. In other studies that used wearables to monitor HR, the Ava bracelet indicated an increase in nightly HR in the luteal phase, but the HRV ratio decreased in the luteal phase, which was different from our study [12]. Changes in HR and HRV may be explained by a decrease in parasympathetic activity after ovulation, in response to increased progesterone [11]. As both BBT and HR data reflected phase-based alterations during the menstrual cycle, it is reasonable that the incorporation of HR data in the algorithm improved the predictive power in our study.

In addition to the Huawei Band 5 used in our study, other wearables, such as the Ava bracelet, Oura ring, in-ear wearable thermometer and OvulaRing (a vaginal biosensor), have been used to record physiological parameters and thereby track menstrual cycles, with machine learning algorithms used to predict the ovulation day and fertile window [10, 12, 14, 30, 31]. Most of these studies exclusively recorded one parameter, body temperature; the Ava bracelet simultaneously monitored additional parameters, including HR, HRV, respiratory rate and skin perfusion. Therefore, it is reasonable that the Ava bracelet demonstrated better performance than other wearables in predicting the fertile window, with an accuracy of 90%, a specificity of 93%, and a sensitivity of 81% [12]. In our study, the accuracy, sensitivity, specificity and AUC of fertile window prediction and menstruation prediction also improved after including HR data collected with the Huawei Band 5. These findings indicate the importance of including various data collected by wearables to establish more reliable prediction models for the fertile window and menstruation. The higher sensitivity and specificity can better distinguish the actual days within the fertile window from other phases, thus helping couples optimize their chance of achieving pregnancy. Surprisingly, the performance of the algorithm did not improve a lot after inclusion data of SDNN and LF/HF ratio, with even lower accuracy, sensitivity and specificity for fertile window prediction among the regular group. This can be possibly explained by the greater dispersion of data of SDNN and LF/HF ratio than BBT and HR in our study (data not shown). Additionally, since the algorithm developed in this study will finally be applied into the software in the smartphone and wearable device, both functionality and simplicity should be taken into consideration. A robust performance based on relatively less features will increase the compatibility and stability of the software. What’s more, the definition of the fertile window in our study was in accordance with the gold standard [1, 32, 33]; therefore, our prediction results were more professional and reliable than those of some other studies [14, 31].

To the best of our knowledge, this is the first study to include both women with regular cycles and those with irregular cycles to predict the fertile window and menstruation with the aid of wearables and machine learning, and the application of algorithms to both groups supports the generalizability of our results. Menstrual trackers such as smartphone apps or wearable sensors which collect women’s health data have become increasingly popular, but most of these studies only included women with regular menstruation cycles [12, 13, 34]. In real-world scenarios, it is impossible for all women to have menstrual cycles that fall within a certain length. A recent study based on 32,595 women found that approximately 13% of participants had actual menstrual cycle lengths that did not fall within the range of 23–35 days. In addition, even in women with a typical 28-day cycle, these apps failed to precisely predict the fertile window [7]. Therefore, the development of more multifaceted and accurate apps to track menstrual cycles is needed for women with irregular menstrual cycles.

Our study showed that data derived from ovulatory women, regardless of their menstrual pattern, have the potential to predict the fertile window and menstruation of women with irregular menstruation cycles. Ovulatory women with a cycle length that either falls within or outside of 25–35 days exhibit similar trends in physiological parameters during the menstrual cycle. The physiological parameters in all phases were similar between regular menstruators and irregular menstruators. These findings establish a theoretical foundation for the development of a prediction model. Accordingly, our preliminary algorithm had a relatively high specificity, 82.90%, and a low sensitivity, 21.00%, for women with menstrual irregularity in fertile window prediction; the algorithm also had a relatively high specificity, 84.40%, and a low sensitivity, 36.30% in menstruation prediction. This indicates a high false-negative rate but a low false-positive rate, which is necessary to detect the actual fertile window and menstruation. We also tried to improve the performance through subgroup analysis based on the cycle length and age of participants, especially for the irregular group. Based on previous literatures, short or long cycles had different pathophysiological mechanisms. A short cycle length could be associated with luteal phase deficiency and a long cycle length could be associated with polycystic ovary syndrome [35, 36]. In addition, age has been reported to be related with physiological parameters during menstrual cycles [37, 38]. These could partly explain why some of the predictive score improved in certain subgroups, while the sample size was too small to draw a solid conclusion. Further studies with larger sample sizes and more variables are needed to optimize these results.

Another strength of our study is that we used a combination of transvaginal sonographic examinations and serum LH levels as our reference standard to determine ovulation. This provides the most reliable method of assessing and predicting ovulatory status. Previous studies estimated ovulation onset via a home urine LH test without resorting to confirmatory ultrasounds or serum hormone tests [12, 14]. Based on prior studies comparing urinary LH kits and endometrium biopsy, false-positive test results are frequent [39]. In addition, ovulation does not always occur one day after the surge in urinary hormone levels [40]. These factors could reduce the precision of ovulation day confirmation. The serial transvaginal follicular ultrasound used in this study serves as the gold standard for identifying the ovulation day [41], thus enhancing the reliability of our prediction algorithms.

### Limitations

This study has some limitations regarding both the device and the algorithm. We only included two parameters, BBT and HR, in the current algorithm. If more variables are measured in the future, the performance of prediction models should be further improved. In addition, the tympanic membrane temperature had to be measured with an ear thermometer and recorded in the app daily, which may have decreased the compliance of participants and the accuracy of data. Our approach would be difficult to apply if a user failed to test or record her temperature in the morning. In the future, a wearable device capable of collecting real-time temperature measurements can improve the performance and generalizability of the machine learning model.

## Conclusions

In conclusion, we demonstrated that BBT and indices related to HR changed in a specific pattern during the menstrual cycle for both regular and irregular menstruators. This pilot study also exhibited the efficacy of machine learning in predicting fertile windows and menstruation in regular menstruators using BBT and HR data collected by a wearable device. We further established the feasibility of fertile window and menstruation prediction among irregular menstruators. Since determining the days when sexual intercourse is more likely to result in successful conception is highly important in clinical practice, it is expected that continuous health data monitoring through a wearable device and a well-established algorithm will help women successfully conceive in the future.

## Supplementary Information

**Additional file 1: FigureS1.** Physiological parameters of the irregular group in the different phases of the menstrual cycle. **Table S1.** Characteristics of all participants. **Table S2.** The relationship between menstrual cycle and physiological parameters in the irregular group. **Table S3.** Accuracy, sensitivity and specificity of different models in fertile window prediction. **Table S4. **Performance of prediction model developed using BBT and HR data of regular group in fertile window prediction in different irregular subgroups. **Table S5.** Performance of prediction model developed using BBT and HR data of regular group in fertile window prediction in different age subgroups. Supplementary Methods.

## Data Availability

The data for this study is available from the corresponding author on reasonable request.

## Abbreviations

AMH: Anti-Müllerian hormone
Apps: Applications
AUC: Area under the receiver-operating characteristic curve
BBT: Basal body temperature
BMI: Body mass index
E2: Estradiol
FSH: Follicle-stimulating hormone
HF: High frequency
HR: Heart rate
HRV: Heart rate variability
LF: Low frequency
LH: Luteinizing hormone
ROC: Receiver operating characteristic
SDNN: Standard deviation of normal-to-normal intervals
