# Supplementary material for: Tracking of menstrual cycles and prediction of the fertile window via measurements of basal body temperature and heart rate as well as machine-learning algorithms
Source: Reprod Biol Endocrinol. 2022 Aug 13;20:118. doi: 10.1186/s12958-022-00993-4 (PMC9375297; doi:10.1186/s12958-022-00993-4)
Supplement: Supplementary file 1 — Additional file 1: FigureS1. Physiological parameters of the irregular group in the different phases of the menstrual cycle. Table S1. Characteristics of all participants. Table S2. The relationship between menstrual cycle and physiological parameters in the irregular group. Table S3. Accuracy, sensitivity and specificity of different models in fertile window prediction. Table S4. Performance of prediction model developed using BBT and HR data of regular group in fertile window prediction in different irregular subgroups. Table S5. Performance of prediction model developed using BBT and HR data of regular group in fertile window prediction in different age subgroups. Supplementary Methods. [file 12958_2022_993_MOESM1_ESM.docx]

Supplementary material

**Figure S1**

**
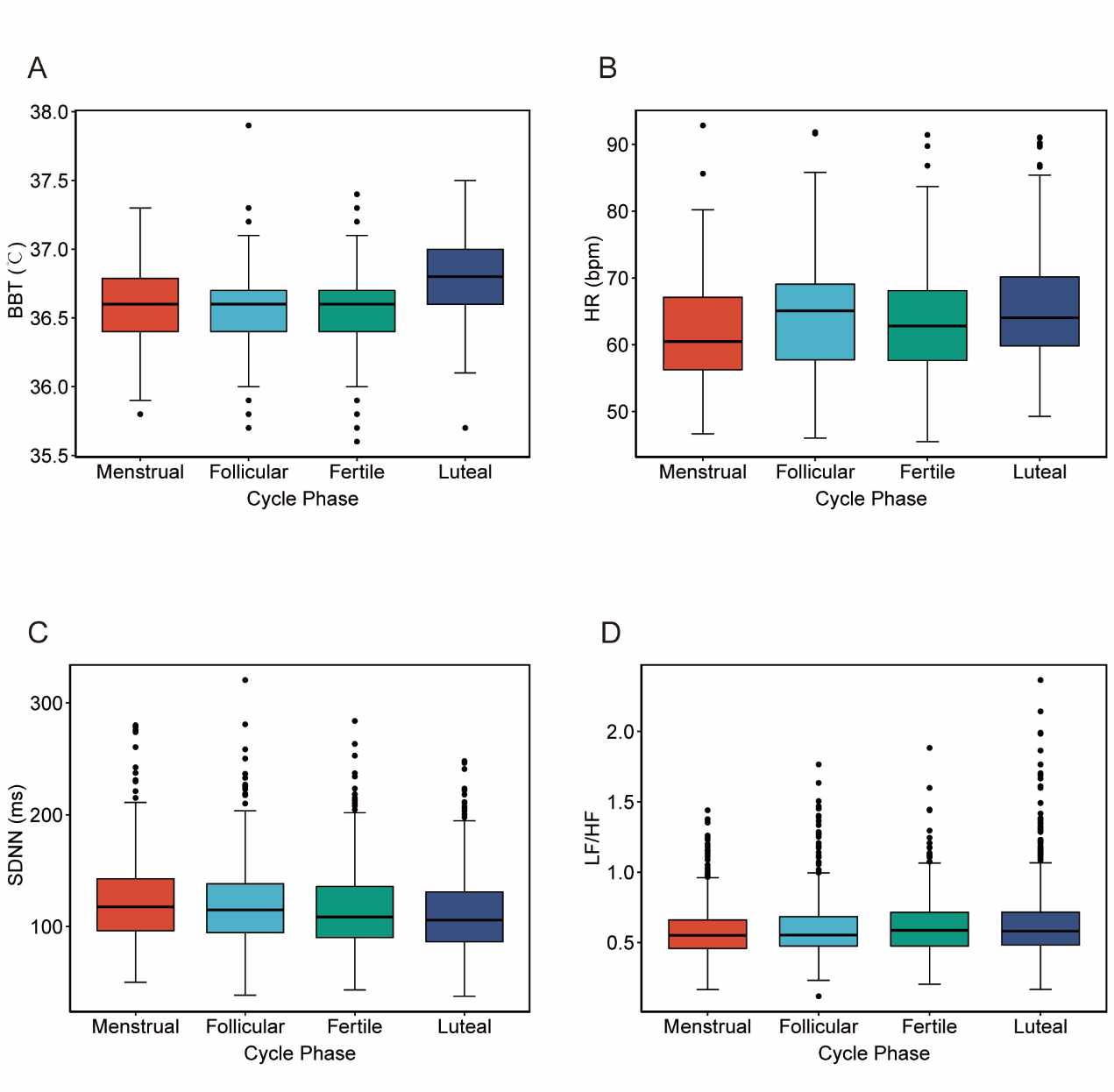
**

**Figure S1. Physiological parameters of the irregular group in the different phases of the menstrual cycle**

Changes in BBT (A), HR (B), SDNN (C) and the LF/HF ratio (D) during the menstrual cycle of irregular menstruators are depicted. The horizontal line represents the medians, boxes represent the values between 25-75%, and lines represent the values between 5-95%. BBT: basal body temperature; HR: heart rate; LF/HF: low frequency /high frequency ratio; SDNN: standard deviation of normal-to-normal intervals.

**Table S1. Characteristics of all participants**

|  | **All** | **Regular** | **Irregular** | ***P*** |
| --- | --- | --- | --- | --- |
|  | **(N=149)** | **(N=99)** | **(N=50)** |  |
|  | **No. (%)** | **No. (%)** | **No. (%)** |  |
| **Age, median (IQR** ^a^**), years** | 31.00 (26.00, 34.00) | 32.00 (26.50, 35.00) | 27.50 (25.00, 31.00) | <0.001 |
| **Age groups, years** |  |  |  | 0.002 |
| 18-24 | 20 (13.4) | 10 (10.1) | 10 (20.0) |  |
| 25-30 | 52 (34.9) | 27 (27.3) | 25 (50.0) |  |
| 31-35 | 52 (34.9) | 40 (40.4) | 12 (24.0) |  |
| 36-45 | 25 (16.8) | 22 (22.2) | 3 (6.0) |  |
| **BMI** ^b^**, median (IQR** ^a^**), kg/m^2^** | 20.82 (19.29, 22.86) | 20.82 (19.33, 22.43) | 20.90 (19.25, 24.11) | 0.472 |
| **BMI groups** |  |  |  | 0.046 |
| <18.5 | 16 (10.7) | 9 (9.1) | 7 (14.0) |  |
| 18.5-23.9 | 108 (72.5) | 78 (78.8) | 30 (60.0) |  |
| ≥24 | 25 (16.8) | 12 (12.1) | 13 (26.0) |  |
| **Marital status** |  |  |  | 0.235 |
| Married | 70 (47.0) | 51 (51.5) | 19 (38.0) |  |
| Single | 77 (51.7) | 47 (47.5) | 30 (60.0) |  |
| Divorced | 2 (1.3) | 1 (1.0) | 1 (2.0) |  |
| **Educational attainment** |  |  |  | 0.373 |
| High school | 5 (3.4) | 4 (4.0) | 1 (2.0) |  |
| College | 68 (45.6) | 41 (41.4) | 27 (54.0) |  |
| Master’s or above | 76 (51.0) | 54 (54.5) | 22 (44.0) |  |
| **Occupation** |  |  |  | 0.245 |
| Unemployed | 36 (24.2) | 22 (22.2) | 14 (28.0) |  |
| Full-time worker | 112 (75.2) | 77 (77.8) | 35 (70.0) |  |
| Part-time worker | 1 (0.7) | 0 (0.0) | 1 (2.0) |  |
| **Duration of menstruation, median (IQR** ^a^**), days** | 6.00 (5.00, 7.00) | 6.00 (5.00, 7.00) | 6.00 (5.00, 6.75) | 0.874 |
| **Menarche, median (IQR** ^a^**), years** | 13.00 (12.00, 14.00) | 13.00 (12.00, 14.00) | 13.00 (12.00, 14.00) | 0.366 |
| **Smokers** | 4 (2.7) | 3 (3.0) | 1 (2.0) | 1 |
| **Consume alcohol** | 35 (23.5) | 24 (24.2) | 11 (22.0) | 0.92 |
| **Previous pregnancy** | 63 (42.3) | 48 (48.5) | 15 (30.0) | 0.048 |
| **Previous childbirth** | 63 (42.3) | 48 (48.5) | 15 (30.0) | 0.048 |
| **AMH** ^c^**, median (IQR** ^a^**), ng/ml** | 4.05 (2.23, 6.21) | 3.40 (2.08, 5.21) | 5.16 (3.68, 7.44) | 0.001 |
| **AMH** ^c^ **groups** |  |  |  | 0.057 |
| <0.7 | 8 (5.4) | 4 (4.0) | 4 (8.0) |  |
| 0.7-7.5 | 118 (79.2) | 84 (84.8) | 34 (68.0) |  |
| >7.5 | 23 (15.4) | 11 (11.1) | 12 (24.0) |  |

^a^ IQR, interquartile range.

^b^ BMI, body mass index.

^c^ AMH, Anti-Müllerian hormone.

**Table S2. The relationship between menstrual cycle and physiological parameters in the irregular group**

| Physiological parameter | BBT ^a, b^ | HR ^a, c^ | Ln(SDNN ^a, d^) | Ln(LF/HF ratio ^a, e^) |
| --- | --- | --- | --- | --- |
| Intercept | 36.60 (0.04) | 62.68 (1.34) | 4.74 (0.04) | -0.56 (0.05) |
| **Cycle phase** |  |  |  |  |
| Menstrual | Reference | Reference | Reference | Reference |
| Follicular | -0.03 (0.01) ^f^ | -0.14 (0.25) | -0.00 (0.01) | -0.00 (0.01) |
| Fertile | -0.03 (0.01) ^f^ | 0.85 (0.26) ^g^ | -0.04 (0.01) ^h^ | 0.04 (0.01) ^h^ |
| Luteal | 0.17 (0.01) ^g^ | 3.37 (0.23) ^g^ | -0.10 (0.01) ^g^ | 0.05 (0.01) ^g^ |
| Follicular | Reference | Reference | Reference | Reference |
| Fertile | 0.00 (0.01) | 1.00 (0.24) ^g^ | -0.04 (0.01) ^h^ | 0.04 (0.01) ^f^ |
| Fertile | Reference | Reference | Reference | Reference |
| Luteal | 0.20 (0.01) ^g^ | 2.52 (0.22) ^g^ | -0.06 (0.01) ^g^ | 0.01 (0.01) |

^a^ Unstandardized beta-coefficient values (standard error) reported with adjusted p values using a Bonferroni correction.

^b^ BBT, basal body temperature.

^c^ HR, heart rate.

^d^ SDNN, standard deviation of normal-to-normal intervals.

^e^ LF/HF ratio, low frequency /high frequency ratio.

^f^ *P* < 0.01.

^g^ *P* < 0.001.

^h^ *P* < 0.05.

**Table S3. Accuracy, sensitivity and specificity of different models in fertile window prediction**

| Physiological parameter | Training dataset (number of subjects/cycles) | Testing dataset (regular group) ^a^ | | | Testing dataset (irregular group) ^b^ | | |
| --- | --- | --- | --- | --- | --- | --- | --- |
|  |  | Accuracy (%) | Sensitivity (%) | Specificity (%) | Accuracy (%) | Sensitivity (%) | Specificity (%) |
| **BBT ^c^ and HR ^d^** | **Regular and irregular group (94/309)** | 86.92 | 67.40 | 92.00 | 77.31 | 35.80 | 85.80 |
|  | **Regular group (74/248)** | 87.89 | 69.80 | 92.70 | 77.31 | 35.80 | 85.80 |
|  | **Irregular group (20/61)** | 84.83 | 62.40 | 90.70 | 78.78 | 35.80 | 87.60 |
| **BBT ^c^, HR ^d^, SDNN ^e^ and LF/HF ratio ^f^** | **Regular and irregular group (94/309)** | 86.76 | 68.20 | 91.60 | 79.62 | 34.60 | 88.90 |
|  | **Regular group (74/248)** | 86.92 | 68.60 | 91.70 | 79.62 | 34.60 | 88.90 |
|  | **Irregular group (20/61)** | 85.31 | 62.40 | 91.30 | 78.36 | 34.60 | 87.30 |

^a^ Testing dataset of regular group including 57 qualified cycles from 15 women.

^b^ Testing dataset of irregular group including 16 qualified cycles from 5 women.

^c^ BBT, basal body temperature.

^d^ HR, heart rate.

^e^ SDNN, standard deviation of normal-to-normal intervals.

^f^ LF/HF ratio, low frequency /high frequency ratio.

**Table S4. Performance of prediction model developed using BBT and HR data of regular group in fertile window prediction in different irregular subgroups**

| Subgroup ^a^ (number of subjects/cycles) | Accuracy (%) | Sensitivity (%) | Specificity (%) |
| --- | --- | --- | --- |
| **< 25 days (5/16)** | 70.76 | 14.30 | 85.70 |
| **> 35 days (20/61)** | 73.21 | 23.80 | 82.80 |
| **< 25 days and > 35 days (25/77)** | 72.51 | 21.00 | 82.90 |

^a^ Testing dataset of irregular group divided by cycle length.

**Table S5. Performance of prediction model developed using BBT and HR data of regular group in fertile window prediction in different age subgroups**

| Group | Subgroup ^a^ (number of subjects/cycles) | Accuracy (%) | Sensitivity (%) | Specificity (%) |
| --- | --- | --- | --- | --- |
| **Regular group** | **≤ 24 years old (2/7)** | 86.74 | 66.70 | 91.70 |
|  | **> 24 years old (13/50)** | 88.09 | 70.30 | 92.80 |
|  | **≤ 30 years old (9/35)** | 86.58 | 64.80 | 92.10 |
|  | **> 30 years old (6/22)** | 90.32 | 78.10 | 93.80 |
|  | **≤ 35 years old (13/50)** | 87.36 | 68.40 | 92.40 |
|  | **> 35 years old (2/7)** | 91.84 | 80.00 | 94.90 |
| **Irregular group** | **≤ 24 years old (6/16)** | 72.96 | 14.20 | 85.10 |
|  | **> 24 years old (19/61)** | 72.79 | 26.00 | 82.30 |
|  | **≤ 30 years old (18/54)** | 73.03 | 19.40 | 83.40 |
|  | **> 30 years old (7/23)** | 72.34 | 28.40 | 82.70 |
|  | **≤ 35 years old (23/70)** | 72.78 | 22.20 | 82.80 |
|  | **> 35 years old (2/7)** | 74.04 | 20.80 | 90.00 |

^a^ Testing dataset of two groups divided by age, respectively.

**Supplementary Methods**

**Data preprocessing step**

The raw data have been preprocessed in the following steps: representative value calculation -> outlier removal -> interpolation -> initial smoothing -> low-pass filtering. Specifically, the raw data were collected in different time-slots within each day and the outliers were removed from the signal. Afterwards, the mean value of the signal was adopted as the representative value for each day and the 30-day window was utilized as the new time series for further analysis. Then, the outliers of 30-day data were further removed and interpolated. Then, for the initial smoothing, the value for each time point was replaced by the mean value of the five-day data preceding the current day (including the data of current day). Finally, the signal was low-pass filtered to remove residual noise.
